# Cross‐sectional and longitudinal associations of caregiving and caregiving stress with caregiver cognitive functioning

**DOI:** 10.1002/alz.093503

**Published:** 2025-01-09

**Authors:** Toni C Antonucci, Kristine J Ajrouch, Laura B. Zahodne

**Affiliations:** ^1^ University of Michigan, Ann Arbor, MI USA; ^2^ Eastern MIchigan University, Ypsilanti, MI USA

## Abstract

**Background:**

Caregiving, both cross‐sectional and longitudinal, is often considered a stressful experience. Less is known about the effect of the caregiving experience or the associated stress on cognitive functioning of the care provider.

**Method:**

Data are drawn from the Social Relations Study (SRS) and the Detroit Area Wellness Network (DAWN) Studies. SRS data are from two face‐to‐face panel data collections in 2005 and 2015, a Detroit area study, focused on social relations among a random representative sample for the Detroit metropolitan area. The DAWN study examined social relations, stress and cognitive performance via a telephone interview during the Covid pandemic, specifically in 2021. Respondents were asked if they were caregiving to a parent, spouse/partner, or (other adult). They were also asked how stressful caregiving has been for them (using a 4 point scale from (1) very stressful to (4) not at all stressful). Cognition was assessed via telephone with multiple measures including the CERAD, Animal Naming, Digit Span F, Digit Span B.

**Result:**

Preliminary findings indicate that people who were caregiving at either time point actually scored higher on cognitive functioning than those who were not; this finding was also evident among people reporting that caregiving was stressful. The same association is evident among those who report feeling stressed by the caregiving experience either previously or contemporaneously. To be precise, they scored higher on all the separate cognitive measures as well as the latent cognitive functioning measure than those who were not stressed by the caregiving experience.

**Conclusion:**

Traditionally caregiving and caregiving stress have been assumed to have a negative effect on the caregiver. These data suggest a more detailed examination of this issue is warranted, especially with respect to cognitive functioning of the caregiver. Additional analyses will examine these findings in greater detail to specify if there are age, education or race health disparities.

